# Clonal Architecture of Secondary Acute Myeloid Leukemia Defined by Single-Cell Sequencing

**DOI:** 10.1371/journal.pgen.1004462

**Published:** 2014-07-10

**Authors:** Andrew E. O. Hughes, Vincent Magrini, Ryan Demeter, Christopher A. Miller, Robert Fulton, Lucinda L. Fulton, William C. Eades, Kevin Elliott, Sharon Heath, Peter Westervelt, Li Ding, Donald F. Conrad, Brian S. White, Jin Shao, Daniel C. Link, John F. DiPersio, Elaine R. Mardis, Richard K. Wilson, Timothy J. Ley, Matthew J. Walter, Timothy A. Graubert

**Affiliations:** 1 Center for Genome Sciences and Systems Biology, Washington University, St. Louis, Missouri, United States of America; 2 The Genome Institute, Washington University, St. Louis, Missouri, United States of America; 3 Department of Genetics, Washington University, St. Louis, Missouri, United States of America; 4 Department of Internal Medicine, Division of Oncology, Washington University, St. Louis, Missouri, United States of America; 5 Siteman Cancer Center, Washington University, St. Louis, Missouri, United States of America; 6 Department of Pathology and Immunology, Washington University, St. Louis, Missouri, United States of America; University of Washington, United States of America

## Abstract

Next-generation sequencing has been used to infer the clonality of heterogeneous tumor samples. These analyses yield specific predictions—the population frequency of individual clones, their genetic composition, and their evolutionary relationships—which we set out to test by sequencing individual cells from three subjects diagnosed with secondary acute myeloid leukemia, each of whom had been previously characterized by whole genome sequencing of unfractionated tumor samples. Single-cell mutation profiling strongly supported the clonal architecture implied by the analysis of bulk material. In addition, it resolved the clonal assignment of single nucleotide variants that had been initially ambiguous and identified areas of previously unappreciated complexity. Accordingly, we find that many of the key assumptions underlying the analysis of tumor clonality by deep sequencing of unfractionated material are valid. Furthermore, we illustrate a single-cell sequencing strategy for interrogating the clonal relationships among known variants that is cost-effective, scalable, and adaptable to the analysis of both hematopoietic and solid tumors, or any heterogeneous population of cells.

## Author Summary

Human cancers are genetically diverse populations of cells that evolve over the course of their natural history or in response to the selective pressure of therapy. In theory, it is possible to infer how this variation is structured into related populations of cells based on the frequency of individual mutations in bulk samples, but the accuracy of these models has not been evaluated across a large number of variants in individual cells. Here, we report a strategy for analyzing hundreds of variants within a single cell, and we apply this method to assess models of tumor clonality derived from bulk samples in three cases of leukemia. The data largely support the predicted population structure, though they suggest specific refinements. This type of approach not only illustrates the biological complexity of human cancer, but it also has the potential to inform patient management. That is, precise knowledge of which variants are present in which populations of cells may allow physicians to more effectively target combinations of mutations and predict how patients will respond to therapy.

## Introduction

Intratumoral heterogeneity is an emerging hallmark of cancer that can be interrogated genome-wide with next-generation sequencing. Critically, sub-populations of tumor cells are organized into hierarchies through clonal evolution. A powerful strategy for studying this population structure is multi-sampling—independently assaying genetic variation at distinct points in time or space and comparing mutation profiles. In particular, whole genome sequencing (WGS) of *de novo* acute myeloid leukemia (AML) has demonstrated genetic evolution between diagnosis and relapse [1], [2], and similar results have been obtained from WGS of paired primary-metastasis samples in breast cancer [3]. Furthermore, whole exome sequencing (WES) of multiple regions within primary tumors has revealed extensive regional heterogeneity in pancreatic [4], hepatocellular [5], and renal [6] carcinomas. Thus, clonal heterogeneity within tumors compounds the biological complexity of human cancers, and a detailed understanding of this is important for clinical genomics.

The ultimate resolution of multi-sampling is single-cell analysis, which is rapidly becoming tractable. For example, Anderson *et al.* have used fluorescence *in situ* hybridization (FISH) to genotype up to five so-called “driver” lesions in individual pediatric acute lymphoblastic leukemia (ALL) cells, which demonstrated a range of clonal architectures (from linear to complex) in different subjects [7]. Jan *et al.*, Potter *et al.*, and Klco *et al.* have reported similar findings using either single-cell allele-specific PCR or amplicon sequencing to assay five to ten clonal markers in *de novo* AML or pediatric ALL [8]–[10]. In broader (genome-wide) analyses, Navin *et al.* and Voet *et al.* have leveraged WGS to call copy number variants (CNVs) in single cells, which they used to reconstruct the phylogenetic history of breast cancer cell lines and primary tumors [11], [12].

In addition to multi-sampling strategies, we and others have reported clonal inference from deep sequencing of individual tumor samples [1], [13]–[15]. Briefly, this approach uses the fraction of sequencing reads calling a specific somatic mutation (i.e., the variant allele fraction, or VAF) to estimate the frequency of that variant in the original sample. Often, large numbers of single nucleotide variants (SNVs) cluster at a common VAF, suggesting the presence of a clonal population at a defined frequency. Analyzing tumors in this way yields specific predictions about the clonal relationships among variants detected in unfractionated samples: 1) the genetic composition of individual clones (groups of SNVs that arose together), 2) the frequency of each clone (proportional to the mean VAF of the corresponding cluster), and 3) a model for how the clonal architecture evolved (clones at lower frequencies descending from those at higher frequencies).

We set out to test these predictions by sequencing single cells from three subjects with an initial diagnosis of myelodysplastic syndrome (MDS), each of whom progressed to secondary AML (sAML). We had previously characterized these subjects by WGS of both MDS and sAML bone marrow as well as matched skin samples, resulting in a call set of several thousand validated somatic mutations in addition to specific models for the clonal architecture of each tumor [14]. In the current study, we used targeted sequencing to genotype >1,900 of these positions in a dozen single cells from each subject. We used SNP array data to quantify the accuracy of single-cell variant calling, and—as reported by others—we observed frequent genotyping errors due to stochastic biases in whole genome amplification (allelic dropout, or ADO) [11], [12], [16]. Nevertheless, while ADO inflated our false negative rate, we maintained a relatively low false positive rate. It was therefore possible to evaluate the major clonal relationships among targeted variants using single-cell sequencing.

Ultimately, the single-cell data strongly supported the major clonal populations predicted from the analysis of bulk tissue, in addition to resolving the clonality of SNVs that were originally ambiguous and suggesting previously unappreciated complexity among rare subclones. Accordingly, our findings validate many of the critical assumptions underlying the inference of tumor clonality from unfractionated samples, in addition to demonstrating a high-throughput approach to single-cell genotyping that provides insight into the clonal architecture of heterogeneous samples.

## Results

### Targeted Sequencing of Single-Cell, Two-Cell, and Unfractionated Samples

We prepared a total of 56 sequencing libraries from whole genome amplified (WGA) single-cell and two-cell sAML samples in addition to non-WGA unfractionated MDS, sAML, and normal (skin) samples (**Table S1**). We used hybridization capture to enrich these libraries for 1,953 somatic SNVs discovered and validated previously in unfractionated samples [14] (**Table S2**). Sequencing yielded 4.1 Gb of de-duplicated data that aligned to targeted loci, resulting in an average depth of coverage of 148× per sample (**Table 1** **, Table S3**). The subject identity corresponding to each sequencing library was confirmed using variant calls at both germline SNPs and targeted somatic SNVs (**Table S4**, **Table S5**). In order to assess the quality of our capture reagent, we compared the VAF distributions of variants in unfractionated MDS and sAML samples to those previously reported [14] (**Figure S1**), finding a strong correlation between these independently-generated datasets (R^2^ = 0.66–0.96).

**Table 1:** Sequencing metrics.

	Total (Mb)	Aligned (Mb)	Aligned (%)	On-Target (%)	Duplicate (%)	On-Target, Unique Coverage (X)
**Sample Average (n = 56)**	408	397	97.3	25.6	29.0	148
**Unsorted Sample Average (n = 14)**	490	487	99.5	35.7	24.0	261
**Single-Cell Sample Average (n = 36)**	382	369	96.6	22.4	30.3	113
**Two-Cell Sample Average (n = 6)**	373	358	96.2	21.0	33.1	94

**Figure 1:**
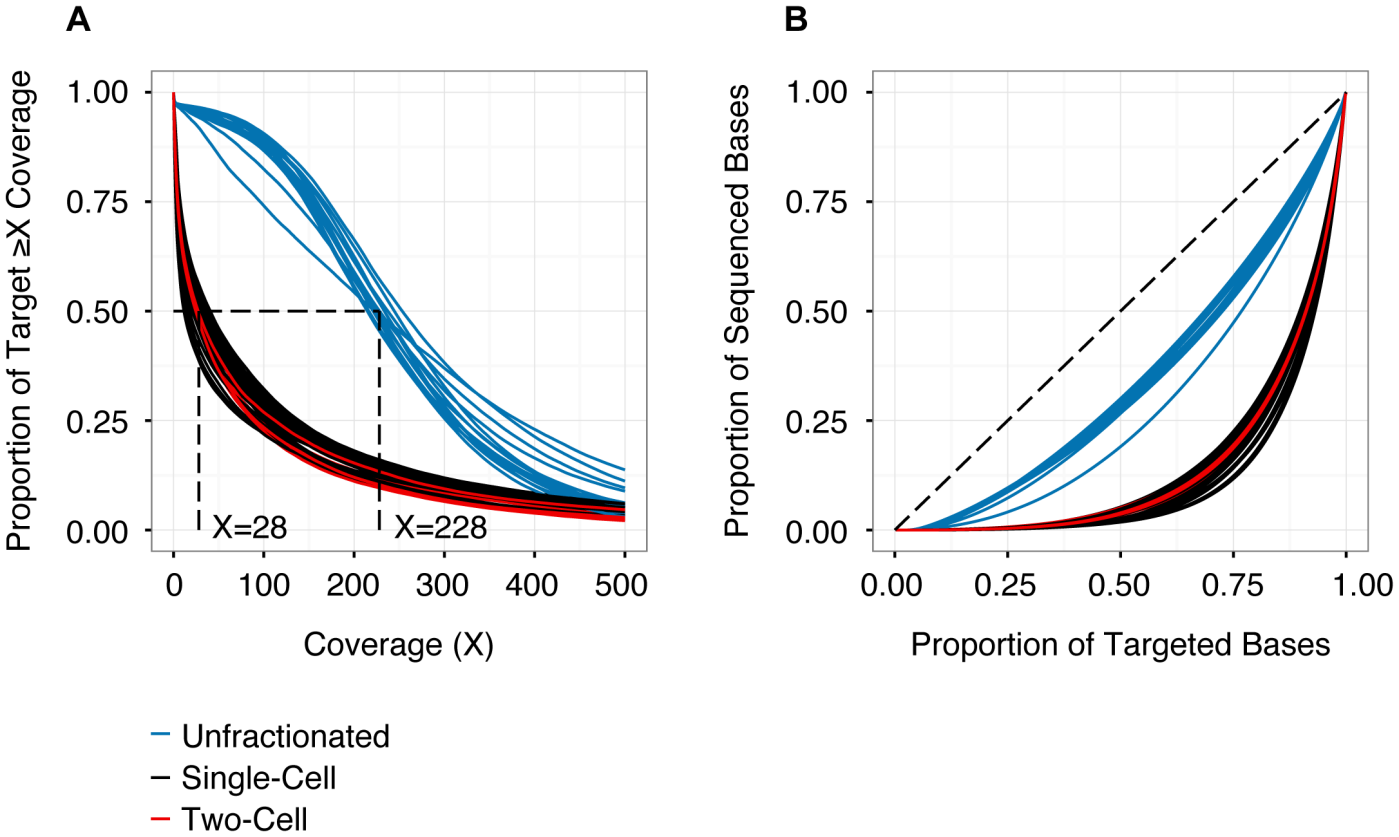
Depth and distribution of coverage for each sequencing library (n = 56). (**A**) Cumulative coverage represented as the proportion of the capture target (y-axis) with read depth greater than or equal to specific coverage thresholds (x-axis). Coverage values are derived from quality-filtered data (de-duplicated, phred-scaled alignment quality ≥10, phred- scaled base quality ≥13). The intersection of each curve with y = 0.5 identifies the median coverage. Higher coverage was obtained for the unsorted samples (median 228×), compared to the single- or two-cell samples (median 28×). (**B**) Lorenz curve detailing uniformity of coverage as proportion of targeted bases versus proportion of sequenced bases. Dashed line (y = x) represents a perfectly uniform distribution of read depth across the capture target. Libraries prepared from WGA samples (single- and two-cells) exhibit significantly less uniform representation, compared to libraries derived from unfractionated material. See **Table 1** and **Table S3** for additional details.

Consistent with previous reports, we observed a number of differences in sequencing performance between WGA libraries and those prepared from unfractionated material [11], [12], [16]. In particular, single- and two-cell libraries had a lower proportion of the capture target covered at any threshold (**Figure 1A**). This was attributable in part to 20% fewer reads obtained from libraries prepared from WGA material (**Table 1**). Furthermore, these libraries had a lower on-target rate (likely driven by locus dropout) and a higher rate of PCR duplicates (i.e., reduced library complexity) (**Table 1**). In addition, single- and two-cell samples had a significantly less uniform distribution of reads across the capture target (**Figure 1B**), again reflecting WGA biases. In aggregate, these technical issues limited callable positions (sites with ≥25× coverage) to approximately 55% of targeted SNVs in single- and two-cell samples (41%–63% for single-cell and 46–53% for two-cell libraries).

### Performance of Variant Calling

**Figure 2:**
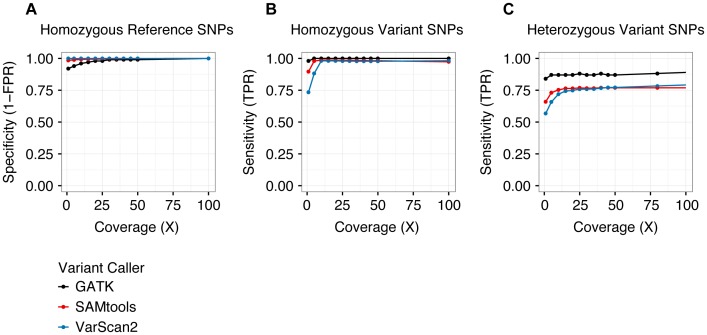
Performance of variant calling. The specificity (**A**) and sensitivity (**B, C**) of three separate variant callers—SAMtools, VarScan2, and GATK—were evaluated by analyzing single-cell variant calls at germline SNPs previously ascertained by Affymetrix SNP arrays [14]. As we have defined true positive and true negative, sensitivity is undefined at homozygous reference positions (there are no true positives) and specificity is undefined at heterozygous and homozygous variant positions (there are no true negatives). Sensitivity and specificity were similar among all three callers at homozygous positions, but GATK demonstrated greater sensitivity at heterozygous sites. Variants were called jointly across all single-cell libraries with the GATK Unified Genotyper utility, whereas variants were called independently for each sample using SAMtools and VarScan2. See **Table 2** and **Table S6** for additional details. TPR: true positive rate. FPR: false positive rate.

To quantify the accuracy of variant calling in single cells, we examined germline (i.e., inherited) SNPs genotyped previously using Affymetrix 6.0 arrays. We evaluated three separate variant callers: SAMtools [17], VarScan2 [18], and the Genome Analysis Toolkit (GATK) Unified Genotyper [19], [20]. With SAMtools and VarScan2, we called variants from individual samples, whereas with GATK, we called variants jointly across all single-cell libraries. At homozygous SNPs, all three callers performed similarly (**Figure 2A, 2B**). However, at heterozygous SNPs (which best approximate targeted SNVs), calling samples jointly yielded a modest benefit in sensitivity, while reducing specificity (**Figure 2C**). Based on these results, we chose to call variants jointly using GATK at sites with ≥25× coverage, and we estimated our sensitivity and specificity for singe-cell variant calling to be 0.88 and 0.98, respectively. As a caveat, benchmarking joint variant calling at germline SNPs (which are present in every cell) potentially overestimates sensitivity to detect subclonal SNVs (which may be present in only a subset of cells). Nevertheless, joint variant calling likely offers a genuine increase in sensitivity, without incurring much cost in specificity, especially when calls are restricted to sites with high coverage.

As shown in **Table 2**, the majority of genotyping errors (assessed at germline SNPs) were false negatives, i.e. failures to detect true non-reference alleles, which resulted in reduced true positive rates (TPRs). These occurred exclusively at heterozygous positions in libraries prepared from WGA material, implicating ADO as the underlying mechanism (approximately equal to the false negative rate, or FNR). This assumption is further supported by the observation that the frequency of homozygous reference calls was similar to that of homozygous variant calls at known heterozygous SNPs (**Figure S2**). ADO is a well-documented limitation of commercial single-cell WGA kits [11], [12], [16]. Nevertheless, although our analysis of germline SNPs demonstrated that single-cell reference allele calls were enriched for false negatives (at heterozygous positions), it also showed that non-reference allele calls were generally accurate (overall false positive rate, or FPR, approximately equal to 0.02). This asymmetry between FNR and FPR was critical for differentiating genuine clonal relationships among targeted SNVs from genotyping errors.

**Table 2:** Performance of variant calling at germline SNPs.

		# of Positions	TPR	FPR	FNR	
					Homozygous Sites	Heterozygous Sites
**UPN461282**	**Average: Unsorted Cells**	17	1.00	0.00	0.00	0.00
	**Average: Single Cells**	9	0.90	0.00	0.00	0.21
	**Average: Two Cells**	8	0.88	0.00	0.00	0.25
**UPN182896**	**Average: Unsorted Cells**	329	0.99	0.00	0.00	0.00
	**Average: Single Cells**	189	0.93	0.01	0.00	0.13
	**Average: Two Cells**	181	0.94	0.01	0.00	0.09
**UPN288033**	**Average: Unsorted Cells**	327	1.00	0.01	0.00	0.00
	**Average: Single Cells**	195	0.92	0.02	0.00	0.13
	**Average: Two Cells**	204	0.98	0.03	0.00	0.04

True Positive (TP): ≥1 non-reference allele called by Affymetrix array, ≥1 non-reference allele called by sequencing.

True Negative (TN): 0 non-reference alleles called by Affymetrix array, 0 non-reference alleles called by sequencing.

False Positive (FP): 0 non-reference alleles called by Affymetrix array, ≥1 non-reference allele called by sequencing.

False Negative (FN): ≥1 non-reference allele called by Affymetrix array, ≥0 non-reference alleles called by sequencing.

True Positive Rate (TPR): TP/(TP+FN) = sensitivity = power.

False Positive Rate (FPR): FP/(FP+TN) = 1-specificity.

False Negative Rate (FNR): FN/(TP+FN) = 1 - sensitivity = type II error.

Finally, we tested whether ADO could be linked to systematic (i.e., locus-specific) effects, or if it was predominantly stochastic. To do this, we compared the rate at which inherited heterozygous SNPs common to all three subjects were called reference in single-cell libraries (**Figure S3**). In general, the dropout rate of a specific locus across single-cell libraries from one subject was not predictive of its dropout rate across single-cell libraries in another (R^2^ = 0.25–0.30), suggesting that ADO was not attributable to strong positional biases.

### Validation of Sample Cellularity

As an additional quality control measure, we asked if the VAF distribution in single cells could be used to infer sample cellularity. In single cells, the true (unobserved) VAF of heterozygous variants is 0.5 (at diploid loci). As shown in **Figure S4, S5, S6**, the VAF distributions in single-cell samples exhibited high variance (ranging from 0 to 1) compared to unsorted samples, reflecting stochastic biases in WGA. However, the mean VAF for each cluster, as well as for germline heterozygous SNPs, was fixed at approximately 0.5. In contrast, in intentionally “cross-contaminated” two-cell samples, the mean VAF of individual clusters (but never germline heterozygotes) dropped to 0.25, the precise dilution expected from the admixture of two cells sharing some, but not all, heterozygous SNVs (**Figure S4**, **Figure S6**). To analyze this further, we modeled these distributions computationally and used maximum likelihood analysis integrating a site-specific error model to assess the probability that each dataset was generated from all possible combinations of two cells. This predicted that >90% of single-cell libraries were derived from true single-cell samples (**Table S7**).

### Assessment of Tumor Clonality

**Figure 3:**
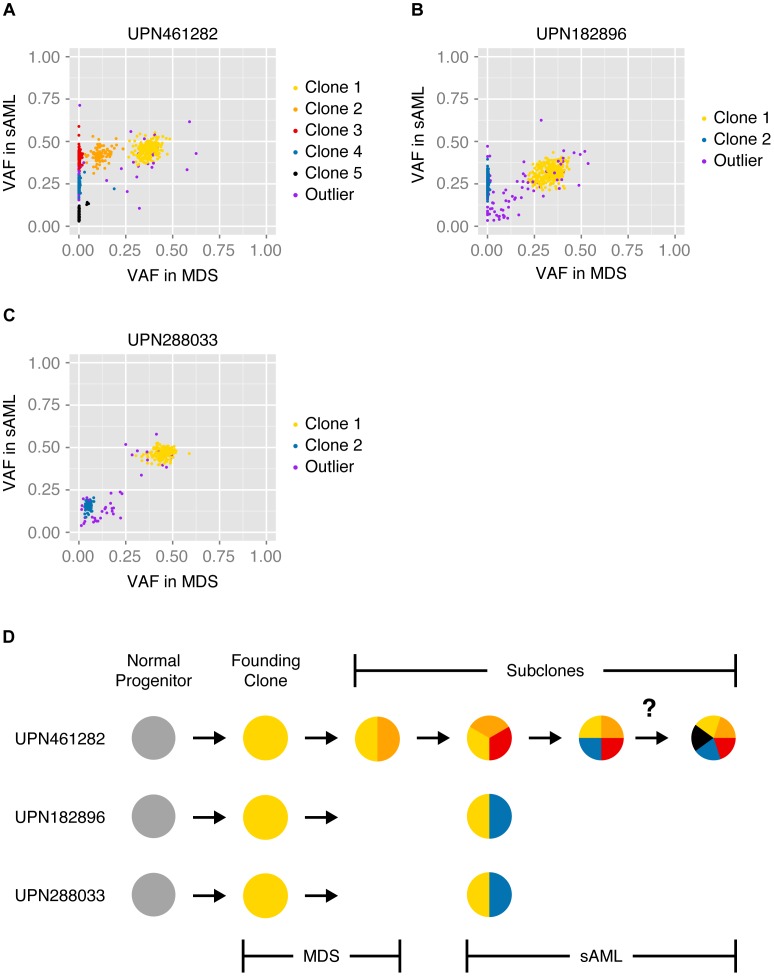
Model of tumor clonality predicted from unfractionated samples. SciClone analysis and cluster assignment of previously validated somatic mutations present in unsorted MDS and sAML bone marrow cells [14], [21]. Variant allele fractions of variants at MDS (x-axis) and sAML (y-axis) predict specific tumor substructure and evolution over time. Color-coded mutation clusters are completely non-overlapping between subjects. (**A**) At sAML diagnosis, UPN461282 was predicted to harbor 10% non-tumor cells in addition to 5 subclones: 1) 6% of cells harboring cluster 1 variants (clone 1), 2) 4% of cells harboring cluster 1 and cluster 2 variants (clone 2), 3) 33% of cells harboring cluster 1, cluster 2, and cluster 3 variants (clone 3), 4) 33% of cells harboring cluster 1, cluster 2, cluster 3, and cluster 4 variants (clone 4), and 5) 14% of cells harboring either cluster 1, cluster 2, cluster 3, and cluster 5 variants or cluster 1, cluster 2, cluster 3, cluster 4, and cluster 5 variants (clone 5). (**B**) At diagnosis with sAML, UPN182896 was predicted to harbor 35% non-tumor cells in addition to 2 subclones: 1) 13% of cells harboring cluster 1 variants (clone 1), and 2) 52% of cells harboring cluster 1 and cluster 2 variants (clone 2). (**C**) At diagnosis with sAML, UPN288033 was predicted to harbor 7% non-tumor cells in addition to 2 subclones: 1) 62% of cells harboring cluster 1 variants (clone 1), and 2) 31% of cells harboring cluster 1 and cluster 2 variants (clone 2). (**D**) Schematic summarizing our initial models of clonal evolution inferred from SciClone analysis—the question mark denotes ambiguity of clone 5 origin in UPN461282.

Previously, we generated WGS data from MDS, sAML and normal samples for each subject in the current study, and we analyzed the VAF distribution of validated somatic mutations to infer the clonal architecture of each tumor [14]. In the current study, we applied SciClone—a variational Bayesian algorithm—to the original WGS data to refine these models [13], [21]. As shown in **Figure 3A–C**, groups of SNVs cluster at distinct frequencies, and we hypothesized that each cluster represented a clonal population of tumor cells. I.e., clustered SNVs were predicted to colocalize within individual cells. Furthermore, we predicted that the population frequency of putative clones was proportional to the mean VAF of the corresponding cluster. Finally, we hypothesized that clones present at successively lower frequencies evolved linearly from clones at higher frequencies, i.e., that these populations were nested. Accordingly, subjects were predicted to be monoclonal (UPN182896) or biclonal (UPN461282, UPN288033) at the time of MDS diagnosis, and harbor two or more clones upon progression to sAML (**Figure 3D**). In addition, our analysis of both unfractionated blood and bone marrow samples indicated that tumor clonality was similar in both compartments (consistent with recent findings in both *de novo* AML and MDS [10], [22]), though UPN288033 had an overall reduction in tumor cells in peripheral blood (**Figure S4, S5, S6**).

**Figure 4:**
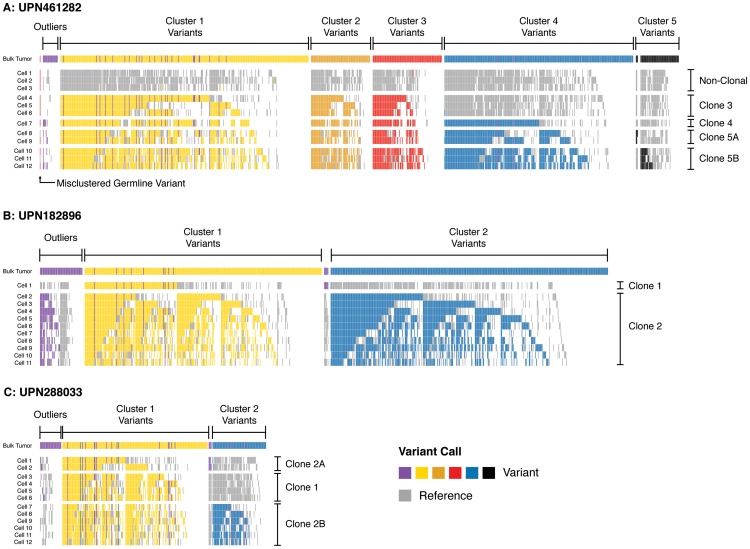
Single-cell mutation profiles. Variant profiles across targeted somatic mutations in single-cell samples (sAML bone marrow) in (**A**) UPN461282, (**B**) UPN182896, and (**C**) UPN288033. Rows display positive and negative variant calls color-coded by mutation cluster for each single-cell sample, and columns indicate specific SNVs somatic at sAML diagnosis. Variants are grouped and color-coded by cluster as predicted from sequencing unfractionated material (uppermost track in each panel). Each cell is grouped by the clone it is inferred to represent. Outlier SNVs (purple) were those which could not be confidently clustered based on bulk sequencing. Here, many of these are merged into predicted clusters based upon their presence/absence in single-cell libraries (i.e., harboring the same pattern as well-defined clones). Positions where reference calls were made are colored grey; positions where no call was made (<25× coverage) are colored white. Pairs of variants that always travel in the same state (reference or variant) likely arose in the same clonal expansion. Pairs of variants that are called together in some cells but not others are likely related by linear evolution. Pairs of variants that are mutually exclusive suggest evolutionary branch points, and were rare. This suggested that variants in subclone 5 in UPN461282 (**A**), and subclone 1 in UPN288033 (**C**) were divided among additional subclones (now 5A/5B, 2A/2B). See **Figure S7** for data presentation with unmodified clone and cluster definitions (derived from bulk sequencing).

To evaluate models of tumor clonality predicted from unfractionated samples, we overlaid tracks of single-cell variant calls on the cluster definitions derived previously (**Figure 4** **, Figure S7**). In general, single-cell mutation profiles strongly supported the existence and composition of the predicted clonal populations. We observed multiple cells from each subject harboring the majority of targeted SNVs, and at least one cell in each subject in which complete clusters of putatively subclonal variants were called reference. Single-cell sequencing thus demonstrated the existence of distinct cells arising at successive points in tumor evolution, in addition to validating our hypothesis that SNVs present at similar VAFs travel together in individual cells.

As shown in **Figure 4**, we observed a significant rate of reference calls (15%, on average) in each cell at sites predicted to be within a mutation cluster. Formally, this could reflect cryptic subclonal heterogeneity, but these positions could not be aggregated into clusters of more than a few variants. Furthermore, mutually exclusive sets of variants that were reference in cells representing the founding clone were recovered in cells corresponding to more mature clones, which would imply an unlikely rate of convergent evolution. Alternatively, these reference calls likely represent stochastic false negatives in each cell. Indeed, the rate of these reference calls was consistent with our estimated FNR (due to ADO) of 0.12. Accordingly, the majority of these reference calls likely reflect ADO (are false negatives), not cryptic population substructure.

While the single-cell genotypes we obtained generally validated our predicted model, they suggested a number of modifications. First, there was ambiguity in our original analysis as to which clone gave rise to cluster 5 variants in UPN461282; this appeared to be a rare subclone that could have emerged from any of its predecessors. The single-cell data unambiguously show that cluster 5 SNVs descended linearly from cluster 4 (i.e., cluster 5 variants always colocalized with cluster 4 variants). Second, approximately 9% of targeted SNVs could not be clustered in our original study, i.e., the clone to which they belonged was ambiguous. For UPN461282 and UPN288033 (for which we had multiple cells representing each clone), we were able to confidently assign 50% of these outliers to specific clones (**Figure 4A, C**). For UPN182896 (for which we only had one cell representing the founding clone), we were only able to recover 35% of outliers (**Figure 4B**).

In addition to resolving the clonality of ambiguous clusters and outliers, the single-cell data identified a small set of variants that were mutually exclusive across multiple cells in each subject—suggesting that a subset of targeted SNVs may in fact represent subclones within the cluster to which they were originally assigned (**Figure 4**). For UPN461282, this occurred among low-frequency cluster 5 variants. Only 20 of the 60 variants we targeted in this cluster were detectable—suggesting that these variants were enriched for false positives or belonged to additional rare subclones not sequenced in the current study—but these 20 appear to be split between two distinct clones. We observed similar evidence of mutually exclusive variant sets (i.e., evolutionary branch points) among the outliers that could be re-clustered in UPN182896 and UPN288033. Again, these potential subclones were small, consisting of only 4–5 SNVs, thus supporting the interpretation that the dominant evolutionary relationship among targeted variants was linear, though a minority of variants may have arisen secondarily to major clonal expansion events.

**Figure 5:**
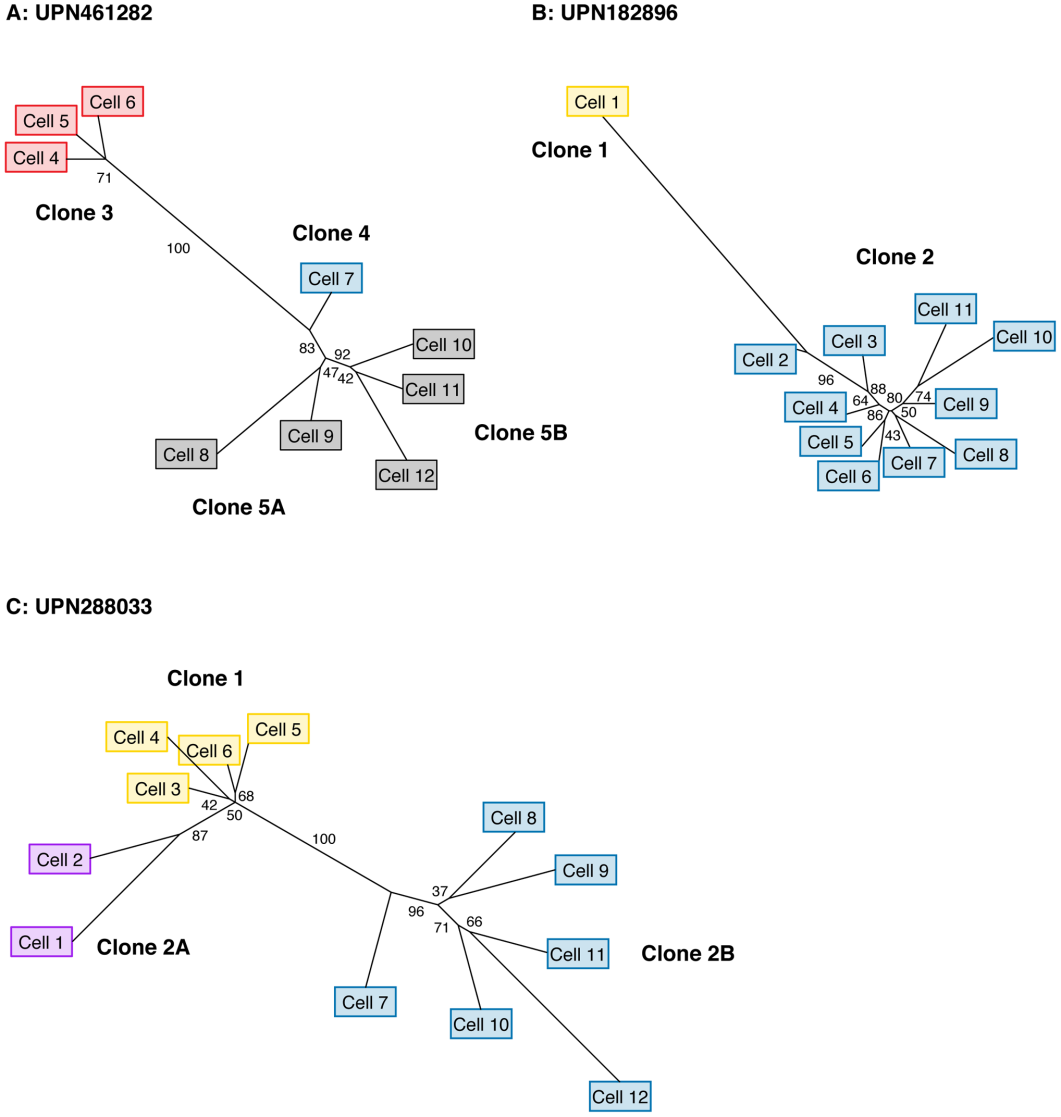
Single-cell reconstruction of tumor phylogeny. Maximum likelihood phylogenetic trees derived from single-cell genotypes at targeted somatic SNVs. (**A**) UPN461282 (**B**) UPN182896 (**C**) UPN288033. Values along edges represent branch support determined by non-parametric bootstrap (n = 1000 iterations). Edges with ≥75% support are considered strongly supported. Cell labels are identical to those in **Figure 4**, and colored based on the presence variant clusters corresponding to the profiles detailed in **Figure 4**.

Finally, we performed phylogenetic analysis to assess tumor clonality based solely on the genetic distance between individual cells (independent of predicted cluster definitions). We used maximum likelihood to reconstruct the phylogenetic tree of each tumor using single-cell genotypes at targeted SNVs (**Figure 5**), which again supported our original model. The major clones ascertained from single-cell mutation profiles were separated by stable branches in each tree. These trees illustrate a generally linear topology, in addition to the branching event within cluster 5 in UPN461282, but they also provide evidence for additional branching events within UPN182896 and UPN288033. We integrated single-cell mutation profiles and trees to assign groups of individual cells to clones; we then compared the frequency of each clone among single cells to our prediction from sequencing unfractionated material, based on the mean VAF of each cluster, and we found a modest but significant correlation (R^2^ = 0.60) (**Figure S8**).

## Discussion

Deep sequencing of unfractionated tumors is a powerful tool for interrogating inter- and intra-tumoral genetic variation [23]. Multiple studies have demonstrated that clonal heterogeneity is a key aspect of cancer biology [13], [15]. These results have validated long-standing models of cancer as an evolutionary process [24], which has clinical implications for the design of effective therapies (selecting targeted agents and predicting response). Indeed, recent work has demonstrated functional heterogeneity among AML subclones [10] as well as prognostic value in detecting subclonal variation in MDS and chronic lymphoid leukemia [25], [26]. Thus, even though the clonal architecture of individual tumors is often strongly implied from sequencing unfractionated samples, a direct assessment of these models and their underlying assumptions is critical.

Here, single-cell analysis of MDS-derived secondary AML samples generally validated predictions from prior analysis of unfractionated samples. The vast majority of SNVs predicted to co-occur in a clonal population were shown to be present in at least one cell, clusters of variants corresponding to subclones were called reference *en bloc* (supporting the predicted evolutionary progression), and the frequency of each clone was correlated (albeit, modestly) with the mean VAF of clusters in unfractionated samples. Nevertheless, the single-cell data suggested specific modifications to the original models. A limited set of variants (n = 3) appear to have been misclustered in the original analysis, 35–50% of outliers could be assigned to clones for the first time, and the ambiguous clonal assignment of clone 5 in UPN461282 was resolved. In addition, approximately 9% of targeted positions (covered in at least one cell) were never called as variants, suggesting that some targeted SNVs were false positives in the original study, or belonged to subclones that were not sampled in this study by chance.

Although most of the variants we targeted were found to colocalize in at least one cell (supporting generally linear evolution), we did observe clusters of variants in each subject that were mutually exclusive (suggesting subclonal branch points). These clusters were typically small (with five or so variants differentiating a putative subclone), but were supported by multiple cells. The strongest evidence for this occurred in a low-frequency subclone in subject UPN461282 (cluster 5 variants). As a class, it is plausible that low-frequency variants may be enriched for complexity, i.e., may tend to be divided among multiple clones, and/or derive from different ancestral populations. Thus, we find that genotyping unfractionated and single-cell libraries are complementary approaches to resolving subclonal complexity. Analysis of unfractionated samples at multiple time points identifies major branches that may not be appreciated at a single time point (e.g., clone 1>clone 2>clone 3 in UPN461282), whereas single-cell genotyping improves the interpretation of low-frequency variants (removing false positive calls and revealing cryptic clonal substructure).

Consistent with our results, recent work by Klco *et al.* has shown that single-cell genotyping supports the tumor clonality predicted from unfractionated *de novo* AML samples [10]. Klco *et al.* used WGA and amplicon sequencing to assay a smaller number of clonal markers (n = 1–3 SNVs per clone, 10 total) across a larger number of single cells (n = 95). This illustrated the utility of a large sample size for accurately estimating clone frequencies from single cells—Klco *et al.* achieved more precise single-cell estimates of variant frequencies that more closely matched estimates from bulk tumors. Alternatively, our analysis of several hundred clonal markers suggested that the secondary AML tumors we analyzed harbored complexity that was not appreciated by bulk analysis, and assaying a small number of variants per clone would not have shown this. Together, the Klco *et al.* study and our own suggest that the clonal architecture of complex tumors is best appreciated through the analysis of a large number of variants across a large number of cells.

Previous reports of single-cell sequencing have already described the primary technical challenges we encountered in this study: 1) locus dropout and non-uniform coverage led to a substantial amount of missing data (positions inadequately covered), and 2) ADO compromised the accuracy of variant calling at heterozygous sites [10]–[12], [16], [27], [28]. Nevertheless, previous studies have generally attempted variant discovery from single-cell sequencing, whereas we sought to genotype a defined set of validated variants, i.e., to understand the clonal relationships among SNVs that were supported by prior knowledge. Alternatively, others have reported analyses of tumor clonality using more accurate single-cell genotyping methods (FISH, allele-specific PCR) [7]–[9], but these lack the throughput required to assay hundreds of variants simultaneously. Therefore, as improvements to WGA technologies are developed (increasing coverage and reducing allelic bias) [16], in addition to more sensitive methods for rare variant detection [29], [30], a capture-based strategy offers an attractive balance of throughput and cost-effectiveness for studying tumor clonality. Accordingly, the approach we have outlined here—integrating both variant discovery in bulk samples, and clonality analysis in single-cells—could be used to confidently localize mutations within clonal hierarchies prior to the initiation of targeted therapies. This has the potential to inform treatment regimens that target complex populations of cells, not just isolated subclones, which may lead to improved patient outcomes.

## Materials and Methods

### Subjects and Samples

All subjects were diagnosed with *de novo* myelodysplastic syndrome (MDS) and progressed to secondary acute myeloid leukemia (sAML) within 32 months. UPN461282: 65 year-old male (refractory anemia with excess blasts and complex karyotype); UPN182896: 75 year-old male (refractory anemia with trisomy 8); UPN288033: 31 year-old female (refractory anemia with excess blasts and normal karyotype). Detailed clinical histories have been reported previously [14]. All subjects provided written informed consent authorizing whole genome sequencing on a protocol approved by the Washington University Office of Human Research Protection.

### SNP Genotyping and Somatic Mutation Discovery in Unfractionated Samples

Affymetrix 6.0 SNP genotyping and WGS of unfractionated normal, MDS, and secondary AML samples were performed as described previously [14]. Somatic mutations were validated by solid phase targeted capture and deep sequencing.

### Isolation and Amplification of Single-Cell DNA

Single vials of cryopreserved bone marrow cells from each subject at sAML diagnosis were thawed, washed in PBS, counted, and adjusted to 7.5 million cells/mL. Single bone marrow cells were deposited into 96 well plates by flow cytometric cell sorting. Additional microtiter plates with two-cells per well were generated to produce intentionally “cross-contaminated” samples. Bivariate plot isolation of single, viable cells was made by forward low angle light scatter and 90 degree light scatter against apex debris and noise, as well as scatter pulse width to isolate single cells from aggregates. This sort decision was applied to a MoFlo cell sorter (Beckman Coulter Inc., Brea, CA) equipped with a Cyclone X-Y deposition instrument, configured to deposit densities of 0–4 cells per well. The coincident cell abort mask was set to be the most stringent, allowing sorted droplets to contain only one target cell with no particles within adjacent droplets.

Cells were sorted directly into extraction buffer; genomic DNA extraction and amplification were carried out using a PicoPlex WGA kit according to the manufacturer's protocol (Rubicon Genomics, Ann Arbor, MI). WGA DNA yield was determined by Qubit fluorometric quantitation (Life Technologies, Carlsbad, CA), and WGA DNA quality was assessed by qPCR.

### Sequencing Library Production and Target Enrichment

Sequencing libraries were prepared from single-cell WGA DNA (n = 12 per subject), two-cell WGA DNA (two cells intentionally deposited in one well, n = 2 per subject), as well as unamplified genomic DNA from unsorted samples—bone marrow and peripheral blood cells (at MDS and sAML diagnosis) and matched normal tissue (skin biopsy) (**Table S1**). Barcoded paired-end Illumina libraries were prepared according to the manufacturer's recommendations (Illumina Inc., San Diego, CA), with the following exceptions: 1) 250–1000 ng of WGA DNA (sorted samples) and 1000–3000 ng of unamplified DNA (unsorted samples) were fragmented using the Covaris E220DNA Sonicator (Covaris Inc., Woburn, MA) to a size range between 100–400 bp; 2) Illumina adapter-ligated library fragments were amplified in four 50 µL PCR reactions for eighteen cycles; 3) Solid Phase Reversible Immobilization (SPRI) bead cleanup was used for enzymatic purification throughout the library process, as well as final library size selection targeting 300–500 bp fragments.

All 56 sequencing libraries were pooled (normalized to 85 ng per library) and hybridized in solution to a custom library of capture oligonucleotides targeting 492,297 bases, according to the manufacturer's protocol (Roche NimbleGen, Madison, WI). Capture baits targeted a total of 1,953 validated somatic single nucleotide variants (SNVs): 872 SNVs from UPN461282, 777 SNVs from UPN182896, and 304 SNVs from UPN288033, as reported previously [14] (**Table S2**). qPCR was used to calibrate flow cell loading concentration and cluster density. Libraries were run on a single lane of an Illumina HiSeq2000, according the manufacturer's recommendations (Illumina Inc., San Diego, CA).

### Bioinformatics Analysis

Illumina reads were de-multiplexed and aligned to the NCBI 37/hg19 reference sequence (GRCh37-lite) using BowTie2 in local mode to allow soft-clipping of WGA adapter sequences [31]. Binary alignment/map (BAM) files were merged and duplicates marked using Picard v1.46 (http://picard.sourceforge.net). Coverage metrics were calculated with GATK v1.2 DepthOfCoverage, with reads filtered for a minimum alignment score of 10 (-mmq10) and a minimum base quality of 13 (-mbq13) [19], [20]. Read pileups were generated for individual samples with the SAMtools v0.1.18 mpileup command using default settings with the following exceptions: 1) base alignment quality (BAQ) computation disabled (-B); 2) minimum alignment score of 10 (-q 10); and 3) minimum base quality score of 13 (-Q 13); 4) maximum read depth of 99999 (-d 99999) [17]. Variants were called from individual sample pileup files with either SAMtools or VarScan v2.3.5 using default parameters [17], [18], or using the GATK Unified Genotyper applied jointly across all single-cell libraries [19], [20]. The identity of each sample was confirmed by variant calls at known germline homozygous SNPs (**Table S4**), as well as individual-specific somatic SNVs (**Table S5**).

### Phylogenetic Analysis

Phylogenetic analysis was performed in R (v3.0.1) using the packages ape [32] and phangorn [33]. Briefly, genetic distances were estimated among all single cells for each subject under a generalized Kimura model [34], and initial trees were derived using a modified neighbor joining algorithm. Likelihood optimization was then used to obtain the maximum likelihood (ML) tree for each subject using a generalized time reversible (GTR) substitution model. Finally, we performed a non-parametric bootstrap on each ML tree to estimate the support for individual branches (n = 1000 iterations).

### Maximum Likelihood Estimation of Sample Cellularity

For each single- and two-cell sample, the number of variant reads at heterozygous loci was modeled as a binomial process with a probability, p, derived from: 1) the variant allele fraction, f, in the original population (putatively 1 or 2 cells), and 2) the cumulative error rate, e, attributable to WGA, library preparation, and sequencing. I.e., for each locus, P(X = k)∼Bin(n,p), where k is the number of variant reads, n is the total read depth, and p is given by p = f(1−e)+(1−f)e. For each mutation cluster, the cumulative likelihood of the observed variant allele counts was calculated using specific VAFs for every possible combination of two clones. A likelihood ratio test using a one-sided chi-square distribution with 1 degree of freedom was then applied to calculate the overall probability that the observed variant allele fraction distribution was generated from a clonally pure (single-cell) or clonally heterogeneous (two-cell) sample. For each subject, the cumulative error rate was estimated for each cluster by calculating the mean VAF at these sites among single-cell samples from the other two subjects (samples expected to be reference at these positions). I.e., since all samples were processed in parallel and run on the same sequencing lane, subjects served as mutual controls for modeling the site-specific error rates intrinsic to WGA, library prep, and sequencing.

## Supporting Information

Figure S1 Correlation of VAFs between array-based and liquid-phase targeted sequencing. Libraries prepared from genomic DNA (without amplification) from unfractionated MDS (left panels) or sAML (right panels) bone marrow cells were enriched for target regions by hybridization capture. The variant allele fraction (VAF) for each targeted SNV determined by array-based (x-axis, previous study [14]) and liquid-phase capture (y-axis, current study) is plotted for each sample. The R^2^ is included for each pair. The VAFs for somatic SNVs are highly correlated between capture reagents.
(PDF)

Figure S2 Genotyping errors at germline heterozygous positions. The average number of genotype calls per library are plotted for each individual and sample type (unfractionated, single- and two-cell) at positions known to be germline heterozygous SNPs (based on Affymetrix arrays). Heterozygous calls (“Het”) therefore represent the correct genotypes at these loci, whereas homozygous reference (“Hom Ref”) or homozygous variant calls (“Hom Alt”) represent a genotyping error due to the loss of a single allele. These errors are rare in unfractionated samples, and—among sorted samples—losses of reference and variant alleles occur at roughly equal rates (two-sided binomial exact test), supporting ADO as the underlying mechanism. Statistical tests comparing proportion of homozygous reference and homozygous variant errors were omitted for UPN461282 due to an inadequate number of genotype observations.
(PDF)

Figure S3 Pairwise comparison of dropout rates among germline heterozygous positions between subjects. The false negative rate (FNR) for each single-cell library was assessed at heterozygous sites common to all three subjects. The R^2^ is included for each pairwise comparison (**A–C**). There appears to be a weak correlation between subjects, but site-specific effects only explain 25–30% of the variance in FNR. I.e., the rate of allelic dropout appears to be predominantly driven by stochastic effects.
(PDF)

Figure S4 VAF distribution for UPN461282 predicted heterozygous somatic mutations among all sequenced samples. (**A**) Unfractionated samples—sAML bone marrow, MDS bone marrow, MDS peripheral blood, and skin—demonstrate the emergence of distinct mutation clusters over time with successively lower mean VAFs. (**B**) The VAF distribution among single cells appears uniform for each cluster, centered on 0.5—except cluster 5, which our analyses suggest was enriched for false positives and composed of at least two mutually exclusive sub-clusters. (**C**) Two-cell experiments show deviations from 0.5 in specific variants—all three clusters in two-cell 1 (suggesting a non-clonal cell mixed with a clone 3 cell), but only cluster 4 in two-cell 2 (consistent with a clone 3 cell mixed with a clone 4 cell). Clone numbers denote the latest mutation cluster observed in a particular cell; e.g. clone 2 harbors mutations from clusters 1 and 2. BM: bone marrow. PB: peripheral blood.
(PDF)

Figure S5 VAF distribution for UPN182896 predicted heterozygous somatic mutations among all sequenced samples. (**A**) Unfractionated samples—sAML bone marrow, sAML peripheral blood, MDS bone marrow, MDS peripheral blood, and skin—demonstrate the emergence of distinct mutation clusters over time with successively lower mean VAFs. (**B**) The VAF distribution among single cells appears uniform for each cluster, centered on 0.5. Cell 12 exhibits less variance than other single cells, suggesting this library was derived from multiple cells (it was excluded from all single-cell analyses). (**C**) Two-cell experiments show no deviations in mean VAF, suggesting two cells belonging to the same clone were sorted in each (clone 2 cells and healthy cells were estimated to constitute 52% and 35% of the sample, respectively). Clone numbers denote the latest mutation cluster observed in a particular cell; e.g. clone 2 harbors mutations from clusters 1 and 2. BM: bone marrow. PB: peripheral blood.
(PDF)

Figure S6 VAF distribution for UPN288033 predicted heterozygous somatic mutations among all sequenced samples. (**A**) Unfractionated samples—sAML bone marrow, sAML peripheral blood, MDS bone marrow, MDS peripheral blood, and skin—demonstrate the emergence of distinct mutation clusters over time with successively lower mean VAFs. (**B**) The VAF distribution among single cells appears uniform for each cluster, centered on 0.5. (**C**) Two-cell experiments show deviations from 0.5 in cluster 2 variants. The mean VAF of cluster 2 in two-cell 2 is diluted near 0.25, consistent with a clone 1 cell mixed with a clone 2 cell. The mean VAF of clusters 1 and 2 in two-cell 1 do not appear to be 0.25 or 0.50, suggesting that more than two cells were sequenced in this library. No non-tumor samples were observed in single- or two-cell samples, but these were only predicted to be present at ∼7%. Here, clone numbers denote the latest mutation cluster observed in a particular cell; e.g. clone 2 harbors mutations from clusters 1 and 2. BM: bone marrow. PB: peripheral blood.
(PDF)

Figure S7 Unedited variant profiles. Variant profiles across targeted somatic mutations in single-cell samples (sAML bone marrow) in (**A**) UPN461282, (**B**) UPN182896, and (**C**) UPN288033. Rows display positive and negative variant calls color-coded by mutation cluster for each single-cell sample, and columns indicate specific SNVs somatic at sAML diagnosis. Variants are grouped and color-coded by cluster as predicted from sequencing unfractionated material (uppermost track in each panel). Each cell is grouped by the clone it is inferred to represent. Outlier SNVs (purple) were those which could not be confidently clustered based on bulk sequencing. Positions where reference calls were made are colored grey; positions where no call was made (<25× coverage) are colored white. Pairs of variants that always travel in the same state (reference or variant) likely arose in the same clonal expansion. Pairs of variants that are called together in some cells but not others are likely related by linear evolution. Pairs of variants that are mutually exclusive suggest evolutionary branch points, and were rare. Clone and variant assignment are derived solely from predictions from bulk sequencing.
(TIF)

Figure S8 Correlation of clone frequencies derived from unfractionated samples and single cells. Previous whole genome sequencing identified 2–5 clusters within the VAF distributions for each subject in the current study [14]. Each cluster was predicted to correspond to a defined subclone at a frequency approximately equal its mean VAF (x-axis). Sequencing 11–12 single-cell libraries and 1–2 two-cell libraries for each subject yielded mutation profiles generally consistent with predicted clones, allowing direct determination of clone frequencies (y-axis).
(PDF)

Table S1 Summary of sample set. Summary of source material for each of the 56 libraries sequenced in this study.
(XLSX)

Table S2 Summary of targeted SNVs. Characteristics of each targeted SNV discovered and validated in the previous study [14]. The right-most 56 columns list the variant allele fraction (VAF) of each variant in every sequenced sample.
(XLSX)

Table S3 Detailed sequencing metrics. Summary of sequencing data generated, aligned, on-target and non-PCR/optical duplicate for each sample.
(XLSX)

Table S4 Identity confirmation at germline homozygous SNPs. Targeted regions included 26 germline homozygous SNPs (ascertained by Affymetrix 6.0 SNP arrays) that differentiated UPN182896 and UPN288033 (data not available for UPN461282). Next-generation sequencing calls verified the sample identity of unfractionated material as well as single-and two-cell samples at these loci (overall true positive rate (TPR) = 0.98).
(XLSX)

Table S5 Identity confirmation at somatic SNVs. For each subject, summary of non-reference calls at positions harboring somatic SNVs in either of the other two subjects. Somatic SNVs were mutually exclusive between subjects, so the expected non-reference call rate is 0, and non-reference calls constitute false positives. These data provided additional verification that each sample was derived from the intended subject (overall false positive rate (FPR)<0.01).
(XLSX)

Table S6 Variant calling performance at germline SNPs. Sample-level data detailing accuracy of next-generation sequencing variant calls compared to Affymetrix array genotypes. True positive (TP): variant allele called by NGS at a site with ≥1 non-reference alleles called by Affymetrix array. True negative (TN): reference alleles called by NGS at a site with 0 non-reference alleles called by Affymetrix array. False positive (FP): variant allele called by NGS at a site with 0 non-reference alleles called by Affymetrix array. False negative (FN): reference allele called by NGS at a site with ≥1 non-reference alleles called by Affymetrix array. True positive rate (TPR): TP/(TP+FN) = sensitivity = power. False positive rate (FPR) = FP/(FP+TN) = 1 – specificity. False negative rate (FNR) = FN/(FN+TP) = 1- sensitivity.
(XLSX)

Table S7 Maximum likelihood analysis. Summary of maximum likelihood analysis of VAF distributions for each single- and 2-cell/well sample. For >90% of single-cell samples, MLE predicts the observed VAFs were derived from a clonally pure sample. The model does not differentiate between 1 cell and 2 cells of the same clone type. It is unlikely that multiple cells were sorted and in 35/36 cases contained only one clone type. Five putative single-cell samples were predicted to be heterogeneous (i.e., they represent the admixture of at least two distinct clones). Four of these involve UPN461282 cluster 5, which 1) appear to have been enriched for false positives, and 2) appear to have been split between at least two independent subclones. The fifth (UPN182896 single-cell 12) appears to be a genuine sorting error, and data from this cell were excluded from all single-cell analyses. Two putative 2-cell/well samples were predicted to be homogenous, which may reflect sorting two cells of the same type (this tumor was largely clone 2 and wild type), or the failure to successfully sort a second cell. MLE Predicted State: most likely two-cell configuration. Expected Clone: state assuming sample was single-cell, belonging to a clone defined by the latest cluster with variant alleles detected. LRT: p-value from one-sided chi-square distribution with one degree of freedom comparing of most likely mixed configuration to most likely unmixed configuration.
(XLSX)
