# Supplementary figures and images for: Clonal Architecture of Secondary Acute Myeloid Leukemia Defined by Single-Cell Sequencing
Source: PLoS Genet. 2014 Jul 10;10(7):e1004462. doi: 10.1371/journal.pgen.1004462 (PMC4091781; doi:10.1371/journal.pgen.1004462)

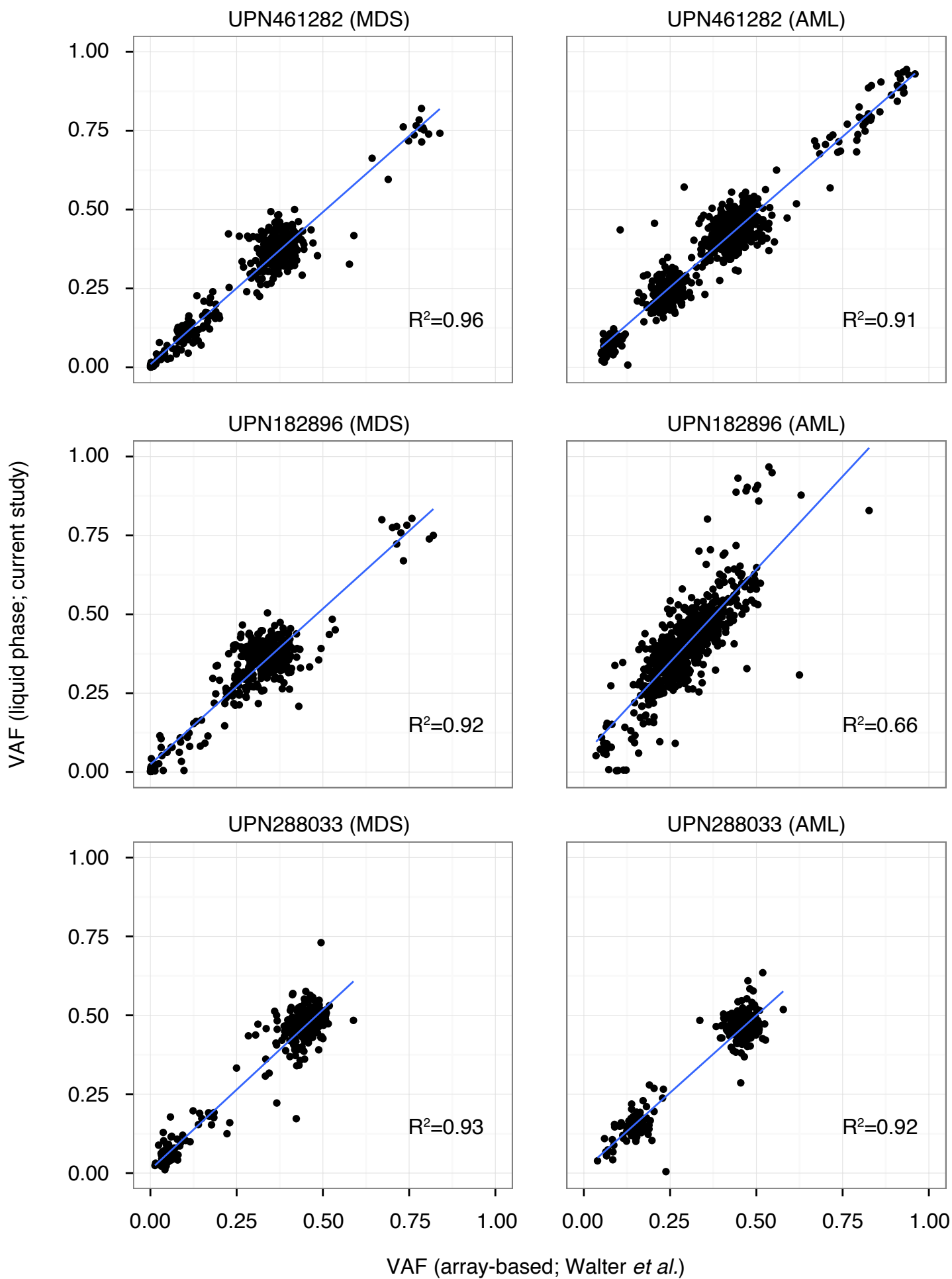

Figure S1

Supplement: Figure S1 — Correlation of VAFs between array-based and liquid-phase targeted sequencing. Libraries prepared from genomic DNA (without amplification) from unfractionated MDS (left panels) or sAML (right panels) bone marrow cells were enriched for target regions by hybridization capture. The variant allele fraction (VAF) for each targeted SNV determined by array-based (x-axis, previous study [14]) and liquid-phase capture (y-axis, current study) is plotted for each sample. The R2 is included for each pair. The VAFs for somatic SNVs are highly correlated between capture reagents. (PDF) [file pgen.1004462.s001.pdf]

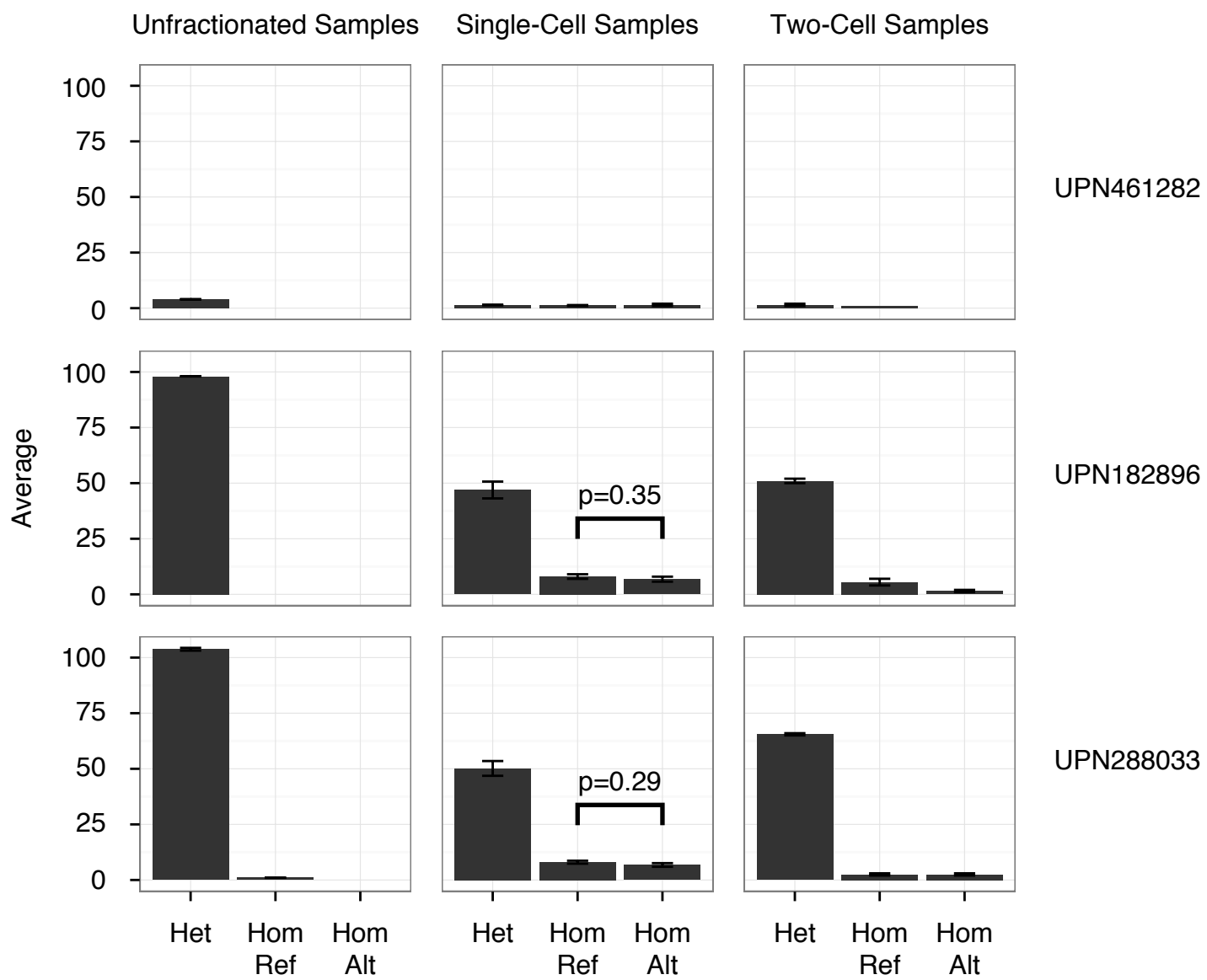

**Figure S2**

Supplement: Figure S2 — Genotyping errors at germline heterozygous positions. The average number of genotype calls per library are plotted for each individual and sample type (unfractionated, single- and two-cell) at positions known to be germline heterozygous SNPs (based on Affymetrix arrays). Heterozygous calls (“Het”) therefore represent the correct genotypes at these loci, whereas homozygous reference (“Hom Ref”) or homozygous variant calls (“Hom Alt”) represent a genotyping error due to the loss of a single allele. These errors are rare in unfractionated samples, and—among sorted samples—losses of reference and variant alleles occur at roughly equal rates (two-sided binomial exact test), supporting ADO as the underlying mechanism. Statistical tests comparing proportion of homozygous reference and homozygous variant errors were omitted for UPN461282 due to an inadequate number of genotype observations. (PDF) [file pgen.1004462.s002.pdf]

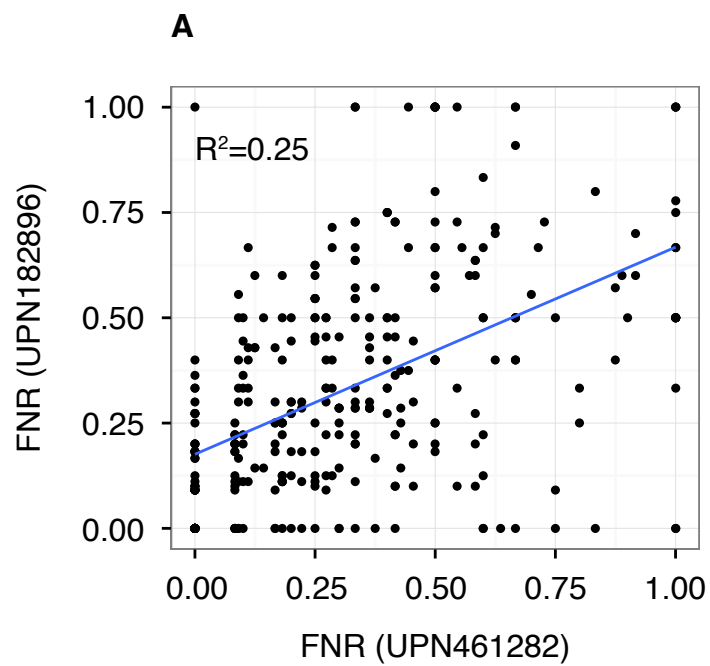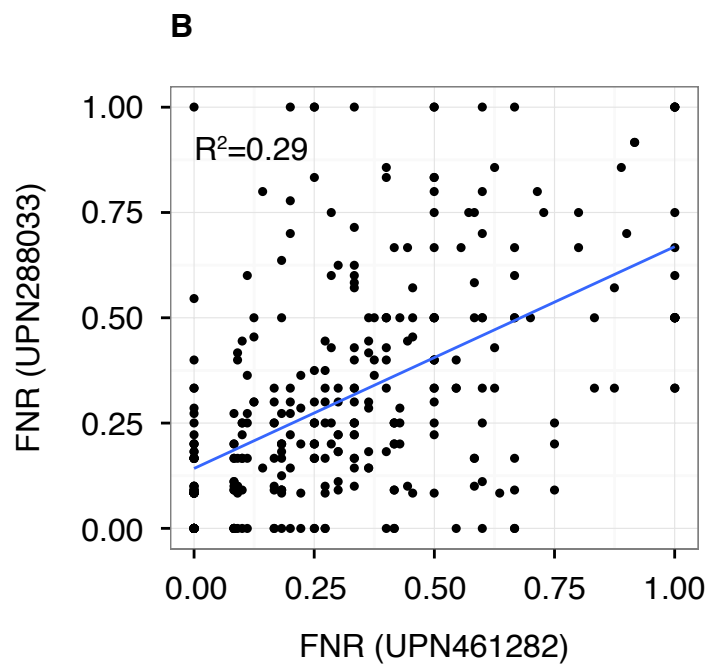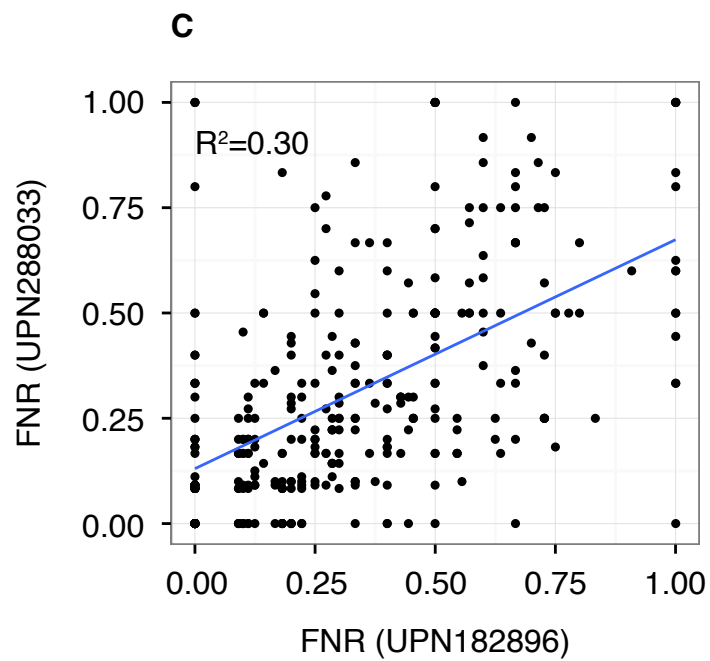

**Figure S3**

Supplement: Figure S3 — Pairwise comparison of dropout rates among germline heterozygous positions between subjects. The false negative rate (FNR) for each single-cell library was assessed at heterozygous sites common to all three subjects. The R2 is included for each pairwise comparison (A–C). There appears to be a weak correlation between subjects, but site-specific effects only explain 25–30% of the variance in FNR. I.e., the rate of allelic dropout appears to be predominantly driven by stochastic effects. (PDF) [file pgen.1004462.s003.pdf]

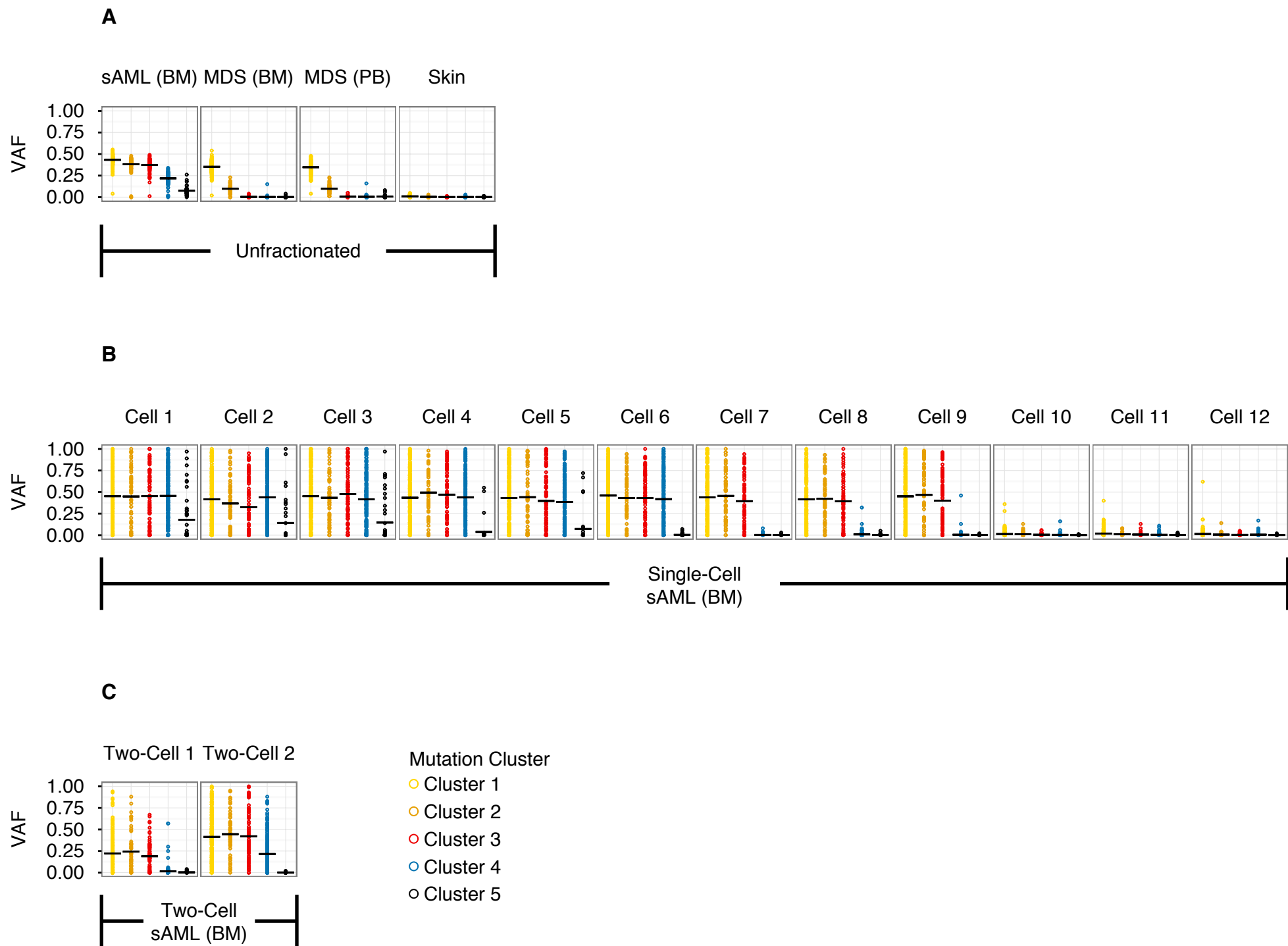

Figure S4

Supplement: Figure S4 — VAF distribution for UPN461282 predicted heterozygous somatic mutations among all sequenced samples. (A) Unfractionated samples—sAML bone marrow, MDS bone marrow, MDS peripheral blood, and skin—demonstrate the emergence of distinct mutation clusters over time with successively lower mean VAFs. (B) The VAF distribution among single cells appears uniform for each cluster, centered on 0.5—except cluster 5, which our analyses suggest was enriched for false positives and composed of at least two mutually exclusive sub-clusters. (C) Two-cell experiments show deviations from 0.5 in specific variants—all three clusters in two-cell 1 (suggesting a non-clonal cell mixed with a clone 3 cell), but only cluster 4 in two-cell 2 (consistent with a clone 3 cell mixed with a clone 4 cell). Clone numbers denote the latest mutation cluster observed in a particular cell; e.g. clone 2 harbors mutations from clusters 1 and 2. BM: bone marrow. PB: peripheral blood. (PDF) [file pgen.1004462.s004.pdf]

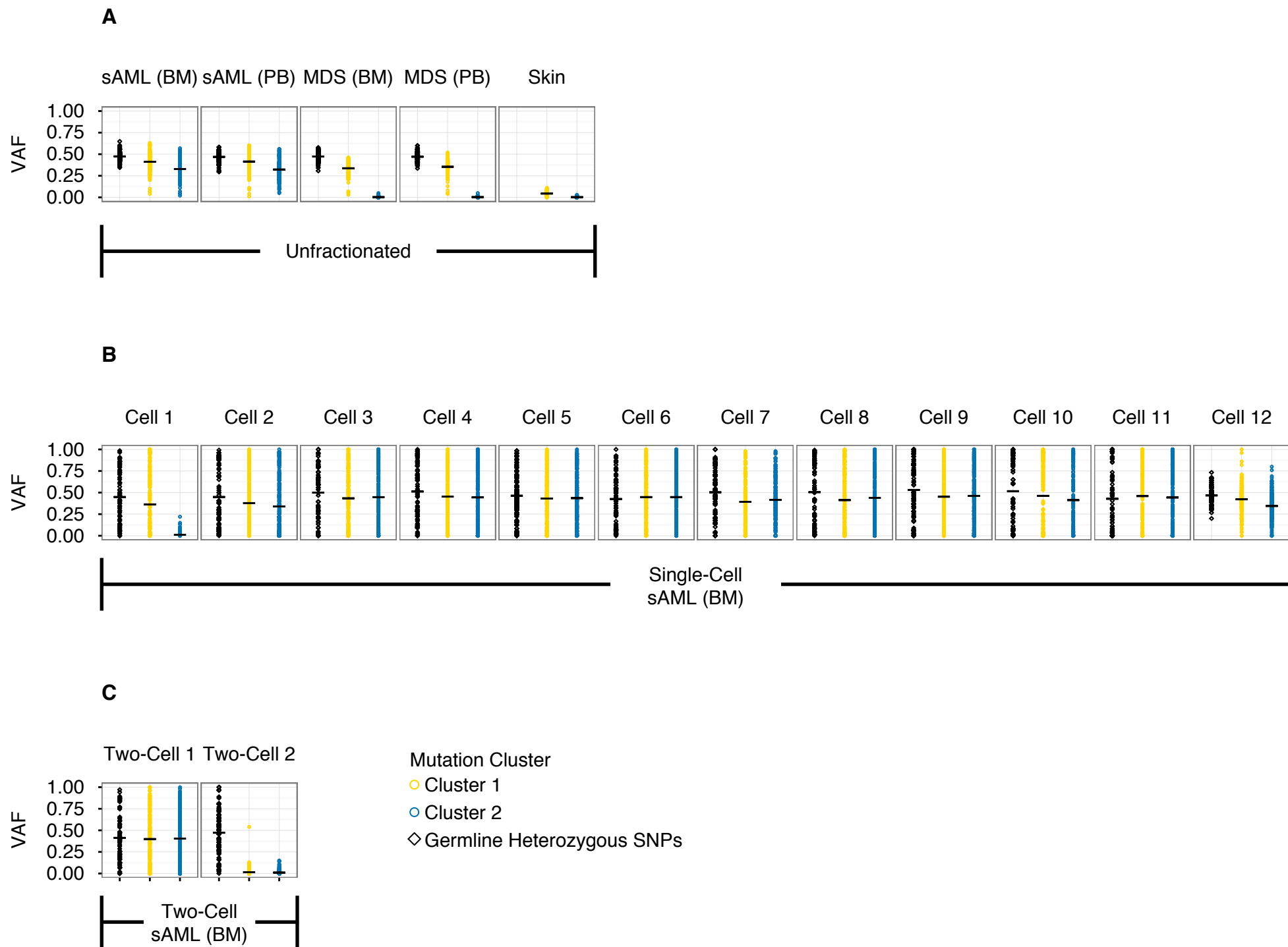

Figure S5

Supplement: Figure S5 — VAF distribution for UPN182896 predicted heterozygous somatic mutations among all sequenced samples. (A) Unfractionated samples—sAML bone marrow, sAML peripheral blood, MDS bone marrow, MDS peripheral blood, and skin—demonstrate the emergence of distinct mutation clusters over time with successively lower mean VAFs. (B) The VAF distribution among single cells appears uniform for each cluster, centered on 0.5. Cell 12 exhibits less variance than other single cells, suggesting this library was derived from multiple cells (it was excluded from all single-cell analyses). (C) Two-cell experiments show no deviations in mean VAF, suggesting two cells belonging to the same clone were sorted in each (clone 2 cells and healthy cells were estimated to constitute 52% and 35% of the sample, respectively). Clone numbers denote the latest mutation cluster observed in a particular cell; e.g. clone 2 harbors mutations from clusters 1 and 2. BM: bone marrow. PB: peripheral blood. (PDF) [file pgen.1004462.s005.pdf]

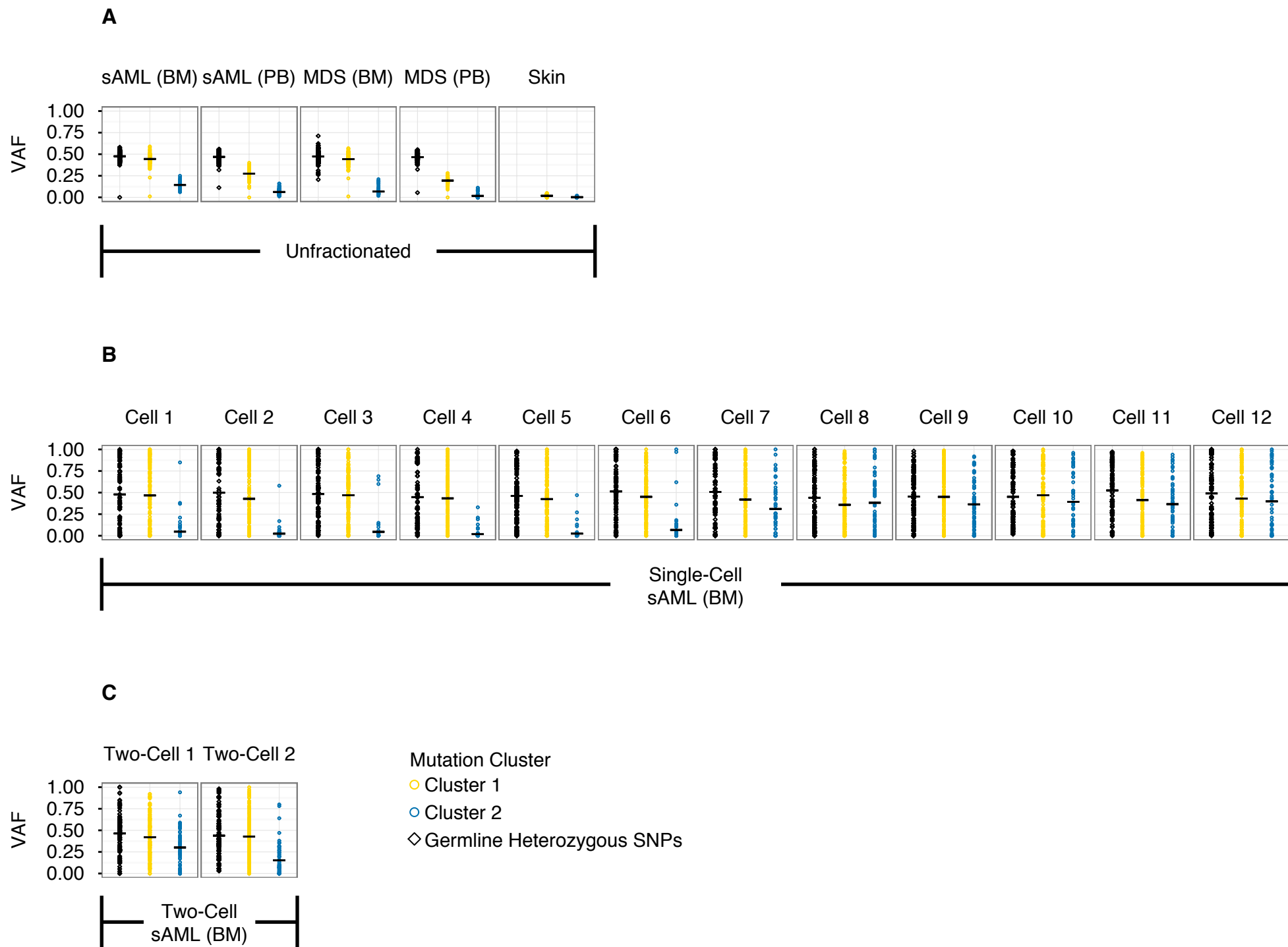

Figure S6

Supplement: Figure S6 — VAF distribution for UPN288033 predicted heterozygous somatic mutations among all sequenced samples. (A) Unfractionated samples—sAML bone marrow, sAML peripheral blood, MDS bone marrow, MDS peripheral blood, and skin—demonstrate the emergence of distinct mutation clusters over time with successively lower mean VAFs. (B) The VAF distribution among single cells appears uniform for each cluster, centered on 0.5. (C) Two-cell experiments show deviations from 0.5 in cluster 2 variants. The mean VAF of cluster 2 in two-cell 2 is diluted near 0.25, consistent with a clone 1 cell mixed with a clone 2 cell. The mean VAF of clusters 1 and 2 in two-cell 1 do not appear to be 0.25 or 0.50, suggesting that more than two cells were sequenced in this library. No non-tumor samples were observed in single- or two-cell samples, but these were only predicted to be present at ∼7%. Here, clone numbers denote the latest mutation cluster observed in a particular cell; e.g. clone 2 harbors mutations from clusters 1 and 2. BM: bone marrow. PB: peripheral blood. (PDF) [file pgen.1004462.s006.pdf]

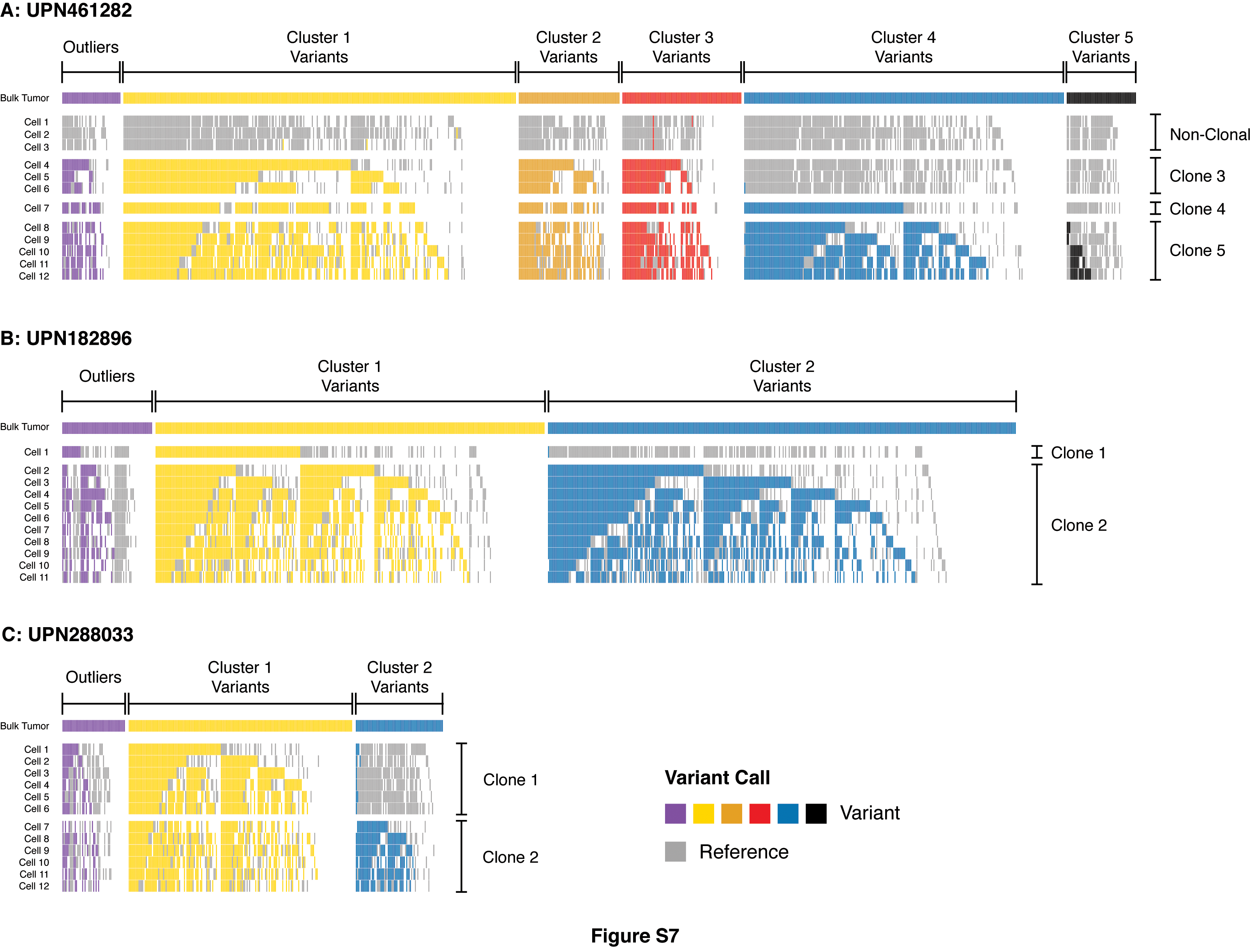

Supplement: Figure S7 — Unedited variant profiles. Variant profiles across targeted somatic mutations in single-cell samples (sAML bone marrow) in (A) UPN461282, (B) UPN182896, and (C) UPN288033. Rows display positive and negative variant calls color-coded by mutation cluster for each single-cell sample, and columns indicate specific SNVs somatic at sAML diagnosis. Variants are grouped and color-coded by cluster as predicted from sequencing unfractionated material (uppermost track in each panel). Each cell is grouped by the clone it is inferred to represent. Outlier SNVs (purple) were those which could not be confidently clustered based on bulk sequencing. Positions where reference calls were made are colored grey; positions where no call was made (<25× coverage) are colored white. Pairs of variants that always travel in the same state (reference or variant) likely arose in the same clonal expansion. Pairs of variants that are called together in some cells but not others are likely related by linear evolution. Pairs of variants that are mutually exclusive suggest evolutionary branch points, and were rare. Clone and variant assignment are derived solely from predictions from bulk sequencing. (TIF) [file pgen.1004462.s007.tif]

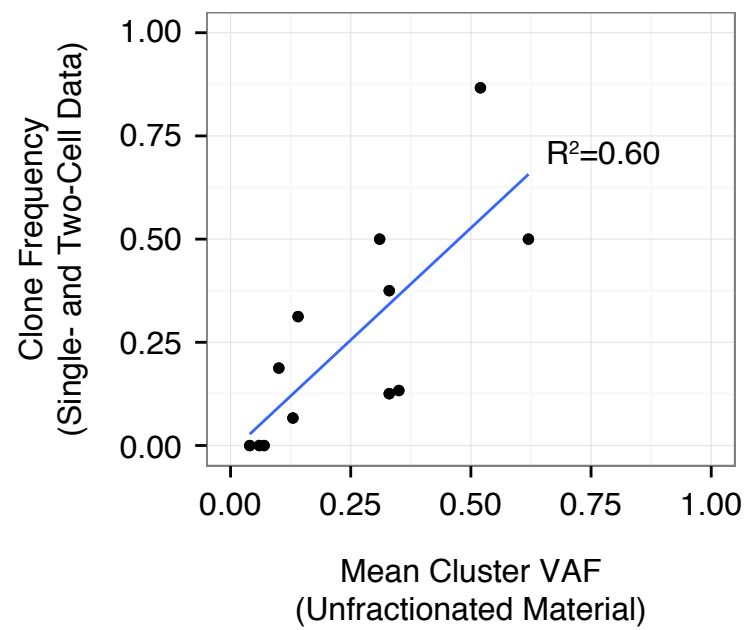

**Figure S8**

Supplement: Figure S8 — Correlation of clone frequencies derived from unfractionated samples and single cells. Previous whole genome sequencing identified 2–5 clusters within the VAF distributions for each subject in the current study [14]. Each cluster was predicted to correspond to a defined subclone at a frequency approximately equal its mean VAF (x-axis). Sequencing 11–12 single-cell libraries and 1–2 two-cell libraries for each subject yielded mutation profiles generally consistent with predicted clones, allowing direct determination of clone frequencies (y-axis). (PDF) [file pgen.1004462.s008.pdf]
